# Concordance and Prognostic Impact of Tumor–Stroma Ratio and Tumor-Infiltrating Lymphocytes in Preoperative Biopsies and Matched Surgical Specimens in Oral Squamous Cell Carcinoma

**DOI:** 10.3390/diagnostics16081202

**Published:** 2026-04-17

**Authors:** Michal Mozola, Michal Herman, Katerina Brachtlova, Jaroslav Michalek, Jana Zapletalova, Zdenek Bednarik, Michal Hendrych, Richard Pink, Peter Tvrdy, Marketa Hermanova

**Affiliations:** 1 Department of Oral and Maxillofacial Surgery, Faculty of Medicine and Dentistry, Palacky University Olomouc and University Hospital Olomouc, 77900 Olomouc, Czech Republic; michal.mozola@fnol.cz (M.M.); michal.herman@fnol.cz (M.H.); katerina.brachtlova@email.cz (K.B.); richard.pink@fnol.cz (R.P.); 2 Department of Clinical and Molecular Pathology, Faculty of Medicine and Dentistry, Palacky University Olomouc and University Hospital Olomouc, 77900 Olomouc, Czech Republic; jaroslav.michalek@fnol.cz; 3 Department of Medical Biophysics, Faculty of Medicine and Dentistry, Palacky University Olomouc, 77900 Olomouc, Czech Republic; ja.zapletalova@upol.cz; 4 First Department of Pathology, Faculty of Medicine, Masaryk University and St. Anne’s University Hospital Brno, 60200 Brno, Czech Republic; zdenek.bednarik@fnusa.cz (Z.B.); michal.hendrych@fnusa.cz (M.H.)

**Keywords:** oral squamous cell carcinoma, tumor–stroma ratio, tumor-infiltrating lymphocytes, pretreatment biopsies, resection specimens, concordance, prognostic impact

## Abstract

**Background/Objectives:** Tumor–stroma ratio (TSR) and tumor-infiltrating lymphocytes (TILs) were suggested as prognostic markers in oral squamous cell carcinoma (OSCC). Identification of markers assessable in preoperative biopsies that could guide treatment planning is of great importance. This study aimed to evaluate the concordance and prognostic impact of TSR and TILs in preoperative biopsies and matched resection specimens of OSCC. **Methods:** This study included 100 patients with OSCC. TSR and stromal TILs were evaluated on hematoxylin and eosin-stained slides of biopsies and paired resection specimens and categorized (into low TSR and high TSR; high TILs and low TILs). The agreement between resections and biopsies, and the prognostic significance and clinicopathological correlations of TSR and TILs, were investigated. **Results:** For TSR, substantial agreement between preoperative biopsies and surgical specimens (kappa correlation coefficient 0.713) was demonstrated. The assessment of TILs showed poor concordance between biopsies and resections (kappa correlation coefficient 0.372). For both biopsies and resections, Cox regression showed an independent negative prognostic impact of low TSR on disease-free, disease-specific, and overall survival. Independent prognostic value of TILs evaluated in biopsies was not found, and the negative prognostic impact of low TILs on disease-free and overall survival was observed only in the main resection specimens. **Conclusions:** TSR evaluated in preoperative biopsies was highly concordant with results in main resection specimens and may provide significant information for OSCC prognostication, risk stratification, and treatment decisions. In contrast, TILs evaluated in biopsies showed poor concordance with main resection specimens and failed to demonstrate prognostic significance.

## 1. Introduction

Oral squamous cell carcinomas (OSCC), representing 90% of all oral malignancies, continue to pose a substantial global health challenge with persistently high mortality rates. With an estimated 390,000 new cases and about 188,000 cancer-related deaths annually, oral cancer is the 16th most common cancer worldwide [1]. Despite advances in diagnostic modalities and therapeutic strategies, the 5-year overall survival rate for OSCC is relatively poor, at about 60%, and survival rates have improved by only 10% between 1980 and 2020 [2,3]. Currently, surgical resection of the primary tumor, with or without neck lymph node dissection, and adjuvant radiotherapy or chemoradiotherapy for patients at high risk of recurrence, remains the standard treatment approach for OSCC [4,5]. Current management, prognostication, and OSCC treatment rely primarily on the American Joint Committee on Cancer (AJCC) tumor-node-metastasis (TNM) staging system [6] supplemented by the standard histopathological parameters such as World Health Organization (WHO) grading, lymphovascular invasion (LVI), perineural invasion (PNI), pattern of invasion (POI), and surgical margin status. Although the 8th Edition of the AJCC enhanced staging performance by incorporating depth of invasion (DOI) and extranodal extension (ENE), considerable heterogeneity persists within each stage regarding biological aggressiveness and recurrence risk [7,8,9]. This limitation is particularly pronounced in early-stage disease, where T-status alone inadequately predicts metastatic potential or recurrence risk [10,11]. The high incidence of locoregional recurrences after primary treatment further demonstrates the limitations of current prognostic models [12,13,14]. Thus, the identification of high-risk patients who could benefit from more aggressive treatment is essential and should be performed as early as possible in the course of the disease [15]. Treatment decisions are typically made during the initial diagnostic work-up when only biopsy material is available [16]. The search for prognostic and predictive markers identifiable in preoperative biopsies with high concordance with findings in surgical resections could contribute to the early identification of high-risk patients and the selection of an appropriate therapeutic procedure. In addition to conventional, predominantly cancer-related histopathological markers, tumor microenvironment (TME)-related biomarkers have gained recognition for their role in disease progression and treatment response [17,18]. Within the TME-related markers, tumor–stroma ratio (TSR) and tumor-infiltrating lymphocytes (TILs) have emerged as promising prognostic factors across various solid malignancies, including head and neck squamous cell carcinomas. In OSCC, studies have demonstrated correlations between high stromal content (low TSR) and decreased patient survival. Similarly, investigations highlight the prognostic significance of TILs, with low densities of stromal TILs associated with worse outcomes [19,20,21,22,23,24,25]. Standardized TIL evaluation methodologies, such as those established by the International Immuno-Oncology Biomarker Working Group (IIBWG), demonstrate consistent results and support their potential as prognostic biomarkers [26,27]. Similarly, standardized and recommended procedures have been introduced for the assessment of the TSR in different tumor types, including head and neck cancer [28,29].

Across numerous studies, analyses of TSR and stromal TILs in OSCC were almost exclusively performed on resection specimens. Concordance between the results obtained from preoperative biopsies and resection specimens has to be established. Reliable assessment of TME-related markers, such as TSR and TILs, in limited samples of preoperative biopsies could provide important information for early identification of high-risk patients and the choice of treatment strategy. This study aims to investigate the concordance of TSR and TILs between diagnostic biopsy and definitive resection specimens in OSCC, thereby validating their potential as early and reliable prognostic markers.

## 2. Materials and Methods

### 2.1. Study Group and Tissue Specimens

This retrospective study included 100 patients with primary OSCC who underwent curative-intent surgical resection at the Department of Oral and Maxillofacial Surgery between March 2015 and February 2023. All involved patients had preoperative biopsies at this institution, and tissue samples from both pretreatment incisional biopsies and matched surgical specimens were available for further analyses. This study was approved by the Ethics Committee of the University Hospital Olomouc, and enrolled patients signed informed consent for treatment and the use of their tissue samples for research purposes. This study included patients with primary OSCC who underwent curative-intent surgery as the initial treatment modality, had no history of squamous cell carcinoma in other body locations, either concurrently or previously, had no distant metastases at the time of diagnosis, had complete clinicopathological and follow-up data, and had tissue samples available. The study did not include patients with a second primary OSCC.

Routinely processed formalin-fixed, paraffin-embedded (FFPE) OSCC preoperative biopsy and surgical resection tissue specimens were retrieved from the archives of the Department of Clinical and Molecular Pathology. Histomorphological data were collected from hematoxylin and eosin (H&E)-stained slides, and all involved cases were histologically reviewed by two dedicated pathologists according to the 5th Edition of the WHO classification of Head and Neck tumors. Restaging was performed according to the 8th Edition of the AJCC Cancer Staging System. For surgical specimens, representative tissue samples with a neoplastic infiltrate of the primary OSCC, including the most invasive tumor front area, were selected for further analysis. For preoperative biopsies, the whole biopsy tissue samples were examined.

Clinical endpoints were the overall survival (OS) defined as time from the date of histopathological confirmation of OSCC diagnosis to death of any cause; disease-free survival (DFS) counted from the date of surgery to the date of any recurrence, local or metastatic, or death of any cause; and disease-specific survival (DSS) calculated from the date of histopathological confirmation of diagnosis to the death of OSCC, or the date of last follow-up for all OS, DFS and DSS.

### 2.2. Assessment of the Tumor–Stroma Ratio (TSR)

**Figure 1:**
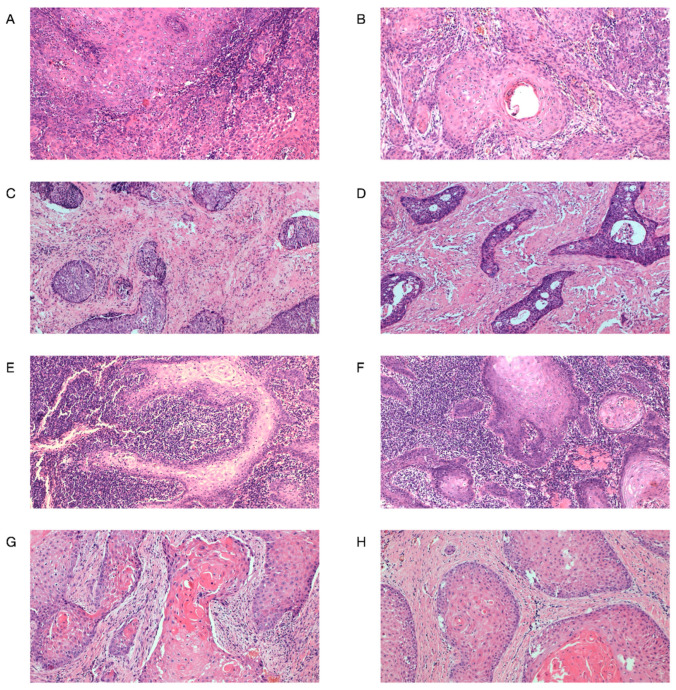
Representative patterns for tumor–stroma ratio and tumor-infiltrating lymphocytes (TILs) in hematoxylin and eosin-stained tissue sections of oral squamous cell carcinoma in matched biopsies and surgical specimens (original magnification ×100): (**A**) high TSR in biopsy; (**B**) high TSR in surgical specimen; (**C**) low TSR in biopsy; (**D**) low TSR in surgical specimen; (**E**) high TILs in biopsy; (**F**) high TILs in surgical specimen; (**G**) low TILs in biopsy; (**H**) low TILs in surgical specimen.

In both biopsies and matched resection specimens, the TSR was visually assessed following the previously described and recommended procedure [28,29]. H&E-stained tissue sections (4 μm-thick) from primary tumors were analyzed by conventional light microscopy. Stromal content was evaluated on whole biopsy tissue sections, and representative tumor tissue sections of corresponding resection specimens were selected. Using the 40× magnification lens, the areas with the highest amount of stroma were identified and used for evaluation. TSR was scored at 100× magnification in areas where both stromal and tumor tissues were present, and tumor cells were visualized on all four sides of the selected field of view, which was assessed. Areas with necrosis, as well as major vascular structures and muscle tissues, were excluded from the stromal compartment. The percentage of stroma was estimated in 10% increments (10%, 20%, 30%, 40%, etc.) per image field. For statistical analysis, the TSR was dichotomized into two categories: high TSR tumors with a proportion of stroma ≤50% and low TSR tumors with a proportion of stroma > 50% within the defined image field (Figure 1A–D). Even if there was only one image field with a stroma-high score, this image field was decisive, and the tumor was classified as low TSR, as proposed [29].

### 2.3. Assessment of Tumor-Infiltrating Lymphocytes (TILs)

In both biopsies and corresponding resection specimens, the evaluation of TILs was performed according to the IIBWG scoring method for the standardized assessment of TILs in solid tumors, in routine H&E-stained tissue sections [26,27]. Briefly, TILs were assessed and reported for the stromal compartment of the tumors and defined as the percentage of the stromal area of the tumor occupied by mononuclear inflammatory cells (lymphocytes and plasma cells) over the total intratumoral stromal area. Areas of necrosis and polymorphonuclear leukocytes were excluded from the scoring. In the main resection specimens, TILs were evaluated within the borders of the invasive tumor in representative selected tissue samples; in biopsies, the total intratumoral stromal area of the tumor was evaluated. An average percentage of TILs assessed in multiple stromal areas was reported not to be focused on hotspots. TILs were semi-quantitatively assessed in percentages as a continuous score (5%, 10%, 20%, 30%, etc.) and categorized into high TILs (≥20%) and low TILs (<20%) (Figure 1E–H).

All histopathological evaluations were performed independently by two observers blinded to clinical data; any discrepancies were resolved by a joint review to obtain a consensus.

### 2.4. Statistical Analysis

The statistical package IBM SPSS Statistics, Version 23 (IBM Corp., Armonk, NY, USA) was used to analyze the data. Student’s *t*-test was used to compare the differences between groups in age. The normality of the distribution of age was tested using the Shapiro–Wilk test. Categorical variables were analyzed by the Chi-square test or Fisher’s exact test. The association between TSR and TIL and demographic and clinical variables was examined using logistic regression. We report the resulting odds ratios (ORs) and their 95% confidence intervals (CIs). The Kaplan–Meier method with the log-rank test was used to analyze survival outcomes. Univariate and multivariate Cox regression analyses (enter method) were carried out to find independent significant predictors of OS, DSS, and DFS. Hazard ratios (HRs) and 95% confidence intervals (95% CIs) were calculated. The concordance between preoperative biopsies and resections was determined using Cohen’s kappa coefficient. Cohen’s kappa and area under the curve from ROC analysis (AUC-ROC) were used to evaluate the interobserver agreement in the initial assessment of TSR and TIL in both biopsies and main resection specimens. Statistical significance was defined as *p* < 0.05.

## 3. Results

### 3.1. Clinicopathological Characteristics and Correlations of TSR and TILs Evaluated in Paired Biopsies and Main Resection Specimens

This study included 100 patients: 69 men (69.0%) and 31 women (31.0%); the average age was 61.8 ± 11.1 years (range: 34–89 years), treated by curative surgery. Tumors were located in the lateral/ventral tongue in 26 patients (26.0%), floor of mouth in 40 patients (40.0%), posterior buccal mucosa in 15 patients (15.0%), and gingiva/alveolar mucosa in 19 patients (19.0%). Regarding the characteristics of the preoperative incisional biopsies, the vertical dimension of the biopsies after tissue processing was ≥5 mm in 76 cases (76.0%) and <5 mm in 24 cases (24.0%). The invasive front of the tumor was histologically demonstrable in 39 (39.0%) of the preoperative biopsy samples. No significant correlation was found between biopsy depth and reaching the invasive front (*p* = 0.477); 11 out of 24 biopsies with a vertical dimension of <5 mm had an invasive front visualized. The clinicopathological information of the patients and their relationship with TSR and TILs scores, both evaluated in preoperative biopsies and in matched resection specimens, are summarized in Table 1 and Table 2.

In both biopsies and main resection specimens, the low TSR was significantly associated with higher grade, pT, lymph node metastasis, higher pTNM stage, PNI, LVI, infiltrative pattern of invasion (POI 4, 5), and local recurrence (*p*-values ranged from ˂0.0001 to 0.046). There was no statistically significant difference between high TSR and low TSR tumors regarding sex. In the main resection specimens, low TSR was significantly associated with lower age of patients, but this association was not demonstrated for low TSR in biopsies. The results are summarized in Table 1.

In both biopsies and main resection specimens, low densities of stromal TILs were significantly associated with higher grade, pT, higher pTNM stage, PNI, and infiltrative pattern of invasion (POI 4, 5) (*p*-values between ˂0.0001–0.008). Low TILs were significantly associated with lymph node metastasis (*p* = 0.005), LVI (*p* = 0.039), and local recurrence (*p* = 0.040) only when evaluated in main resection specimens, and these associations were not observed if TILs were evaluated in biopsies. The results are summarized in Table 2.

Univariate logistic regression analysis showed a significant association of low TSR and pT3 and 4 (resections: OR 3.214; 95% CI 1.270–8.137; *p* = 0.014; biopsies: OR 3.459; 95% CI 1.116–10.721; *p* = 0.032), pTNM stage III and IV (resections: OR 5.296; 95% CI 1.666–16.84; *p* = 0.005; biopsies: OR 6.033; 95% CI 1.296–28.07; *p* = 0.022), PNI (resections: OR 5.624; 95% CI 2.165–14.61; *p* = 0.0004; biopsies: OR 4.218; 95% CI 1.425–12.48; *p* = 0.009), infiltrative pattern of invasion (POI 4, 5) (resections: OR 18.97; 95% CI 4.170–86.25; *p* = 0.0001; biopsies: OR 8.462; 95% CI 1.819–39.36; *p* = 0.006), LVI (resections: OR 2.635; 95% CI 1.036–6.700; *p* = 0.042; biopsies: OR 3.544; 95% CI 1.207–10.41; *p* = 0.021), local recurrence (resections: OR 4.832; 95% CI 1.888–12.37; *p* = 0.001; biopsies: OR 5.900; 95% CI 1.875–18.56; *p* = 0.002), and lymph node metastasis–pN+ (resections: OR 3.022; 95% CI 1.169–7.815; *p* = 0.023). Low TILs were significantly associated with pT3 and 4 (resections: OR 5.373; 95% CI 2.280–12.66; *p* = 0.0001; biopsies: OR 3.249; 95% CI 1.189–8.878; *p* = 0.022), pTNM stage III and IV (resections: OR 4.176; 95% CI 1.730–10.083; *p* = 0.001; biopsies: OR 5.429; 95% CI 1.485–19.85; *p* = 0.011), grade 3 (resections: OR 2.812; 95% CI 1.044–7.575; *p* = 0.041; biopsies: OR 4.286; 95% CI 1.497–12.27; *p* = 0.007), PNI (resections: OR 5.750; 95% CI 2.229–14.83; *p* = 0.0003; biopsies: OR 4.815; 95% CI 1.771–13.09; *p* = 0.002), infiltrative pattern of invasion (POI 4, 5) (resections: OR 11.281; 95% CI 4.318–29.48; *p* = 0.0001; biopsies: OR 7.781; 95% CI 2.127–28.46; *p* = 0.002), local recurrence (resections: OR 3.154; 95% CI 1.347–7.382; *p* = 0.008; biopsies: OR 3.454; 95% CI 1.297–9.198; *p* = 0.013), lymph node metastasis–pN+ (resections: OR 3.388; 95% CI 1.430–8.029; *p* = 0.006).

Additionally, the interobserver agreement in the assessment of TSR and TILs was evaluated. Kappa statistics showed the almost perfect interobserver agreement in the initial assessment of TSR in biopsies (Cohen’s kappa 0.827), TSR in main resection specimens (Cohen’s kappa 0.899), and TILs in main resection specimens (Cohen’s kappa 0.880); substantial interobserver agreement was demonstrated for TILs evaluated in biopsies (Cohen’s kappa 0.775). AUC-ROC of 0.948 (95% CI: 0.885–1.000) for TSR, and 0.940 (95% CI: 0.885–0.994) for TILs evaluated in main resection specimens; 0.904 (95% CI: 0.801–1.000) for TSR, and 0.867 (95% CI: 0.764–0.970) for TILs evaluated in biopsies also showed good interobserver agreement.

### 3.2. Concordance of TSR and TILs Evaluated in Paired Biopsies and Main Resection Specimens

In preoperative biopsies, the tumors were evaluated as high TSR in 83 (83.0%) and low TSR in 17 (17.0%) cases. In matched resection specimens, the tumors were evaluated as high TSR in 73 (73.0%) and low TSR in 27 (27.0%) cases. The evaluation of TSR showed substantial concordance between preoperative biopsies and resection specimens, with a Cohen’s kappa of 0.713. Out of 100 evaluated matched biopsies and resections, 73 cases were high TSR and 17 cases were low TSR in both tissue samples (i.e., 90% agreement). In 10 cases, a high TSR tumor in the biopsy was evaluated as low TSR in the main resection specimens. Thus, the significant shift to low TSR was demonstrated between TSR evaluated in biopsies and in main resection specimens (*p* = 0.002). The results are summarized in Table 3.

In preoperative biopsies, low TILs were demonstrated in 22 (22.0%) cases, and high TILs were displayed in 78 (78.0%) OSCC cases. In resections, low TILs were demonstrated in 46 (46.0%) cases, and high TILs were demonstrated in 54 (54.0%) cases. The evaluation of TILs showed poor concordance between preoperative biopsies and resection specimens with a Cohen’s kappa of 0.372. Out of 100 evaluated matched biopsies and resection specimens, 19 cases had low TILs and 51 cases had high TILs in both tissue samples (i.e., 70% agreement). In 27 cases, high TIL tumors in the biopsy were evaluated as low TILs in resection specimens; in 3 cases, low TIL tumors in the biopsy were evaluated as high TILs in resection specimens. Thus, the significant shift to TILs low was demonstrated between TILs evaluated in biopsies and resection specimens (*p* < 0.0001). The results are summarized in Table 3.

### 3.3. Survival Analysis

**Figure 2:**
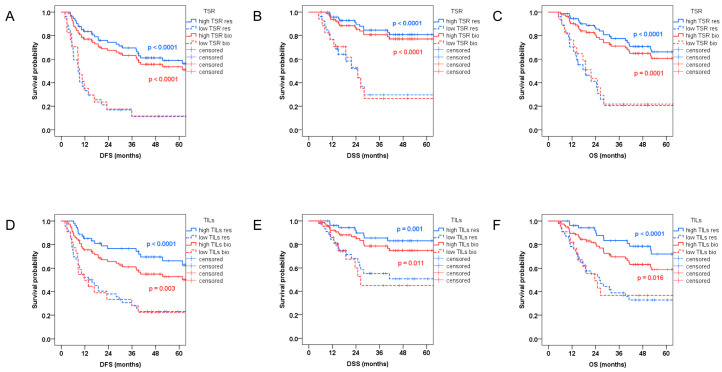
Kaplan–Meier analysis of disease-free survival (DFS), disease-specific survival (DSS), and overall survival (OS) according to tumor–stroma ratio, and levels of tumor-infiltrating lymphocytes (TILs) in biopsies and resections. (**A**–**C**): DFS, DSS, and OS in high TSR versus low TSR tumors in biopsies and resections; (**D**–**F**): DFS, DSS, and OS in high TILs versus low TILs tumors in biopsies and resections.

The median follow-up time was 36 months (range, 5–111 months). During the follow-up, 31 patients (31.0%) displayed local recurrence, and 41 patients (41.0%) died. In Kaplan–Meier analysis, OSCC patients with low TSR tumors showed significantly shorter DFS, DSS, and OS according to TSR assessed in biopsies and main surgical specimens, respectively (Figure 2A–C). Similarly, OSCC patients with low TIL tumors showed significantly shorter DFS, DSS, and OS according to TILs evaluated in both biopsies and main surgical specimens (Figure 2D–F). To judge a potentially biased comparison of survival, as the groups differ substantially in tumor size and nodal status, Kaplan–Meier analyses were separately performed for pT1 + pT2 and pT3 + pT4 groups, and for pN0 and pN+ groups. In both groups, pT1 + pT2 and pT3 + pT4, low TSR and low TILs tumors showed significantly shorter DFS according to TSR in both biopsies and main resection specimens, and TILs in main resection specimens. In pT1 + pT2, low TSR tumors displayed significantly shorter DSS and OS according to TSR in both biopsies and main resection specimens. In the pN+ group, low TSR and low TILs tumors showed significantly shorter DFS and OS according to TSR in both biopsies and main resection specimens, and TILs in main resection specimens.

Univariate Cox regression analysis showed that TSR was prognostic for DFS (biopsies: HR 3.687; 95% CI 1.995–6.814; *p* < 0.0001; resections: HR 5.087; 95% CI 2.859–9.053; *p* < 0.0001), DSS (biopsies: HR 4.909; 95% CI 2.195–10.98; *p* = 0.0001; resections: HR 6.555; 95% CI 2.940–14.62; *p* < 0.0001) and OS (biopsies: HR 3.542; 95% CI 1.778–7.056; *p* = 0.0003; resections: HR 5.504; 95% CI 2.875–10.54; *p* < 0.0001) evaluated in both biopsies and matched resection specimens, with a higher risk of recurrence or death for low TSR OSCC (results summarized in Table 4).

The negative prognostic impact of low TILs was demonstrated for DFS (biopsies: HR 2.420; 95% CI 1.332–4.398; *p* = 0.004; resections: HR 3.824; 95% CI 2.121–6.897; *p* < 0.0001), DSS (biopsies: HR 2.759; 95% CI 1.217–6.251; *p* = 0.015; resections: HR 3.879; 95% CI 1.676–8.975; *p* = 0.002) and OS (biopsies: HR 2.261; 95% CI 1.144–4.469; *p* = 0.019; resections: HR 4.010; 95% CI 2.061–7.802; *p* < 0.0001) evaluated in both biopsies and matched resection specimens (results summarized in Table 4).

In the multivariate Cox regression analysis, TSR remained an independent prognostic for DFS (biopsies: aHR 2.766; 95% CI 1.393–5.492; *p* = 0.004; resections: aHR 4.403; 95% CI 2.241–8.648; *p* < 0.0001), DSS (biopsies: aHR 2.921; 95% CI 1.220–6.994; *p* = 0.016; resections: aHR 5.010; 95% CI 2.032–12.35; *p* = 0.0005) and OS (biopsies: aHR 2.171; 95% CI 1.040–4.530; *p* = 0.039; resections: aHR 4.104; 95% CI 2.000–8.420; *p* = 0.0001) in both biopsies and matched resection specimens, with a higher risk of recurrence or death for low TSR OSCC. For TILs, the independent prognostic significance was confirmed for DFS (HR 2.118; 95% CI 1.043–4.302; *p* = 0.038) and OS (HR 2.210; 95% CI 1.022–4.776; *p* = 0.044) only for resection specimens, with no significant independent prognostic impact for TILs evaluated in paired biopsies (results summarized in Table 4).

## 4. Discussion

Oral squamous cell carcinoma remains a major public health concern due to its aggressive behavior and poor prognosis in a significant proportion of cases [1]. Diagnosis of OSCC is followed by preoperative staging routinely based on clinical and imaging modalities to determine the risk category and choice of the most appropriate treatment strategy. Pretreatment biopsy remains indispensable in the diagnosis of OSCC. However, expanding the importance of biopsy to include a role in prognostic and risk stratification of OSCC patients is desirable, and the identification of histopathological prognostic markers applicable in the evaluation of preoperative biopsies, which could potentially influence clinical treatment decisions, is needed. In OSCC, features related to the TME and host immune response have been extensively studied over the last decade. TSR and TILs displayed prognostic significance if evaluated in main resection specimens [19,20,21,22,23,24,25]. There is a very limited number of studies evaluating these markers in preoperative biopsies [30,31], and the concordance between biopsies and surgical specimens, their prognostic significance, and feasibility for early risk stratification need to be evaluated.

Based on these findings, an analysis of TSR and TILs has been performed in 100 cases of OSCC preoperative biopsies and paired surgical specimens. Our study demonstrated 90% concordance in the assessment of TSR in preoperative biopsies and matched surgical resection specimens, and kappa statistics showed substantial agreement between them. All disagreement cases (10%) had a high TSR evaluated in the biopsy and a low TSR evaluated in the corresponding surgical specimen. Thus, the high TSR category assessed in the preoperative biopsy does not exclude the identification of the defined stroma-high spot in the corresponding surgical specimen and the final classification of the tumor in the low TSR category. On the contrary, all cases assessed as low TSR in the biopsy were consistently evaluated the same way in the paired surgical specimen, and identification of the defined stroma-high area in the preoperative biopsy following the standardized methodology may represent a reliable tool for the final classification of the tumor in the low TSR category. TSR assessment and early identification of low TSR tumors in preoperative biopsies could therefore serve as an additional tool for identifying high-risk patients who might benefit from a more aggressive therapeutic and surgical approach. Our results are in agreement with the conclusions of the studies by Knief et al. and Bello et al. that, so far, are the only ones to have analyzed the concordance between TSR in preoperative biopsies and paired surgical resection specimens of OSCC and oral tongue squamous cell carcinoma (OTSCC), respectively [30,31]. Regarding the evaluation of TSR, the substantial agreement between preoperative biopsies and surgical specimens was demonstrated in the study by Knief et al., which showed concordant results in 88.5% of cases. Bello et al. also showed a good level of agreement in the evaluation of TSR between OTSCC biopsies and resections with 71.4% accuracy. Among head and neck cancers, a similar analysis was performed for laryngeal squamous cell carcinoma, and the TSR evaluated on biopsy was concordant with the TSR assessed in the paired surgical specimen in 88% of cases [32,33]. Moreover, in our study, the prognostic significance of TSR evaluated in both pretreatment biopsies and surgical specimens was shown, and an independent prognostic effect of TSR was confirmed for both in the multivariate analysis. Low TSR tumors displayed significantly worse survival outcomes in terms of disease-free, disease-specific, and overall survival.

On the contrary, for TILs, no significant agreement was achieved between the evaluation in preoperative biopsies and surgical resections. So far, the only published study that analyzed TILs in paired biopsies and OTSCC resections reached similar results and demonstrated low concordance between biopsies and resections in the assessment of TILs following the IIBWG methodology [31]. Additionally, our study demonstrated independent prognostic significance only for TILs evaluated in the main resection specimens, with a negative prognostic effect of low TILs in terms of disease-free and overall survival. Thus, the independent prognostic significance of TILs evaluated in preoperative biopsies was not demonstrated.

A possible explanation lies in the fact that TSR is based on the assessment of the amount of stroma in one spot with the highest stroma content, following the recommended methodology [29]. This standardized methodology, based on identifying a single representative stroma-high field, makes it suitable also for biopsy evaluation, and even one stroma-high field determines the TSR classification. Unlike that, the standardized IIBWG methodology for evaluating TILs determines the average percentage of TILs evaluated in multiple stromal areas, not to be focused on hotspots [27]. Thus, the stromal TILs displayed in the preoperative biopsy do not reflect significant variability in the lymphocytic host response in different parts of the tumor. TILs in biopsies may not display the real picture of the tumor, as they provide only a snapshot of the stromal TILs infiltration in the area of the tumor from which the biopsy was taken. Moreover, this poor concordance may not be solely attributed to general tumor heterogeneity, but likely reflects a combination of the true spatial heterogeneity of the tumor immune microenvironment and sampling limitations of incisional biopsies, particularly if the biopsy fails to capture a representative tumor area, especially the invasive front, where TILs are expanded.

Assessment of stromal features is limited by the quality of the biopsy. Reliable assessment of stromal features may require deeper biopsies, recommended ≥5 mm deep after tissue processing [31], where there is a higher probability of capturing the invasive front of the tumor, where tumor–stroma interactions are best represented. Most incisional biopsies (76%) in the presented study reached the required depth, but in a significant proportion of them, the margin of the invasive front was not histologically captured. Shallow biopsies (<5 mm deep) displayed the invasive front in superficially invasive tumors and may also be useful for the estimation of these features. There is also the question of whether taking multiple biopsies could improve the representation of the tumor’s immune microenvironment. This procedure could theoretically reduce sampling error and better capture the variability of TILs density, but its routine use is problematic for ethical and medical reasons. Routine multi-site biopsy must be balanced against additional patient morbidity, greater tissue trauma and wound burden, possible bleeding and delayed healing, increased healthcare costs, potential delay of definitive treatment, and the theoretical risk of biopsy-related tumor seeding. From a practical and ethical standpoint, routine multiple biopsies solely for biomarker assessment may therefore be difficult to justify. A more realistic strategy may be to optimize the initial biopsy by targeting a representative, adequately deep area that includes the invasive front whenever anatomically and clinically feasible.

Either way, the quality of the biopsy represents one of the main limitations of this study, and the biopsy may not necessarily be representative of the entire tumor. In the context of the presented results, only the identification of a stroma-high spot in the preoperative biopsy has a significant negative prognostic value, with potential individualized implications for the management of OSCC patients. Further limitations of the study include its retrospective design, which may lead to selection bias due to the exclusion of some cases for missing tissue samples or incomplete clinicopathological and follow-up data, and the relatively small sample size.

## 5. Conclusions

In conclusion, this study showed that the assessment of TSR in preoperative biopsies shows substantial agreement with TSR evaluated in OSCC resection specimens. Furthermore, an independent prognostic value of TSR assessed in biopsies as well as resections was demonstrated, and low TSR was significantly associated with worse disease-free, disease-specific, and overall survival. Thus, the evaluation of TSR in preoperative biopsies might provide early information on OSCC prognostication, risk stratification, and treatment decisions. On the contrary, assessment of stromal TILs infiltration showed poor concordance between preoperative biopsies and surgical resections, and the independent negative prognostic value of low TILs was demonstrated only if evaluated in the main resection specimens. Based on these results, the evaluation of TILs in preoperative biopsies does not offer valuable prognostic information in OSCC, and this marker does not represent an efficient early risk stratification tool.

## Figures and Tables

**Table 1:** Clinicopathological characteristics of the study cohort and their correlations with tumor–stroma ratio in pretreatment biopsies and matched surgical specimens.

Characteristics		Total	Tumor–Stroma Ratio in Biopsy			Tumor–Stroma Ratio in Resection		
			High TSR	Low TSR	*p*-Value *	High TSR	Low TSR	*p*-Value *
		*n *(%)	*n *(%)	*n *(%)		*n *(%)	*n *(%)	
		100 (100)	83 (83.0)	17 (17.0)		73 (73.0)	27 (27.0)	
Age (years), mean (SD)		61.8 (11.1)	62.8 (10.7)	57.0 (12.1)	0.051	63.5 (10.5)	57.1 (11.6)	**0.010**
Sex	F	31 (31.0)	25 (30.1)	6 (35.3)	0.674	23 (31.5)	8 (29.6)	0.857
	M	69 (69.0)	58 (69.9)	11 (64.7)		50 (68.5)	19 (70.4)	
Grade	G1	20 (20.0)	20 (24.1)	0 (0.0)	**0.005**	20 (27.4)	0 (0.0)	**0.005**
	G2	54 (54.0)	46 (55.4)	8 (47.1)		38 (52.1)	16 (59.3)	
	G3	26 (26.0)	17 (20.5)	9 (52.9)		15 (20.5)	11 (40.7)	
pT	pT1	26 (26.0)	26 (31.3)	0 (0.0)	**0.011**	25 (34.2)	1 (3.7)	**0.011**
	pT2	28 (28.0)	23 (27.7)	5 (29.4)		20 (27.4)	8 (29.6)	
	pT3	21 (21.0)	17 (20.5)	4 (23.5)		14 (19.2)	7 (25.9)	
	pT4	25 (25.0)	17 (20.5)	8 (47.1)		14 (19.2)	11 (40.7)	
pN	pN0	49 (53.8)	44 (58.7)	5 (31.3)	**0.046**	40 (61.5)	9 (34.6)	**0.020**
	pN+	42 (46.2)	31 (41.3)	11 (68.8)		25 (38.5)	17 (65.4)	
pTNMstage	I	21 (21.0)	21 (25.3)	0 (0.0)	**0.003**	20 (27.4)	1 (3.7)	**0.006**
	II	18 (18.0)	16 (19.3)	2 (11.8)		15 (20.5)	3 (11.1)	
	III	17 (17.0)	16 (19.3)	1 (5.9)		13 (17.8)	4 (14.8)	
	IV	44 (44.0)	30 (36.1)	14 (82.4)		25 (34.2)	19 (70.4)	
PNI	0	69 (69.0)	62 (74.7)	7 (41.2)	**0.006**	58 (79.5)	11 (40.7)	**0.0002**
	1	31 (31.0)	21 (25.3)	10 (58.8)		15 (20.5)	16 (59.3)	
LVI	0	71 (71.0)	63 (75.9)	8 (47.1)	**0.036**	56 (76.7)	15 (55.6)	**0.038**
	1	29 (29.0)	20 (24.1)	9 (52.9)		17 (23.3)	12 (44.4)	
POI	1–3	46 (46.0)	44 (53.0)	2 (11.8)	**0.002**	44 (60.3)	2 (7.4)	**<0.0001**
	4, 5	54 (54.0)	39 (47.0)	15 (88.2)		29 (39.7)	25 (92.6)	
Adjuvant therapy	No	52 (52.0)	47 (56.6)	5 (29.4)	**0.041**	43 (58.9)	9 (33.3)	**0.023**
	Yes	48 (48.0)	36 (43.4)	12 (70.6)		30 (41.1)	18 (66.7)	

* Significant results are indicated in bold.

**Table 2:** Clinicopathological characteristics of the study cohort and their correlations with tumor-infiltrating lymphocytes in pretreatment biopsies and matched surgical specimens.

Characteristics		Total	TILs in Biopsy			TILs in Resection		
			Low TILs	High TILs	*p*-Value *	Low TILs	High TILs	*p*-Value *
		*n *(%)	*n *(%)	*n *(%)		*n *(%)	*n *(%)	
		100 (100)	22 (22.0)	78 (78.0)		46 (46.0)	54 (54.0)	
Age (years), mean (SD)		61.8 (11.1)	59.2 (10.0)	62.5 (11.4)	0.224	60.5 (10.8)	62.9 (11.4)	0.290
Sex	F	31 (31.0)	4 (18.2)	27 (34.6)	0.141	13 (28.3)	18 (33.3)	0.585
	M	69 (69.0)	18 (81.8)	51 (65.4)		33 (71.7)	36 (66.7)	
Grade	G1	20 (20.0)	1 (4.2)	19 (24.4)	**0.001**	4 (8.7)	16 (29.6)	**0.004**
	G2	54 (54.0)	9 (40.9)	45 (57.7)		24 (52.2)	30 (55.6)	
	G3	26 (26.0)	12 (54.5)	14 (17.9)		18 (39.1)	8 (14.8)	
pT	pT1	26 (26.0)	1 (4.5)	25 (32.1)	**0.007**	5 (10.9)	21 (38.9)	**0.001**
	pT2	28 (28.0)	6 (27.3)	22 (28.2)		10 (21.7)	18 (33.3)	
	pT3	21 (21.0)	4 (18.2)	17 (21.8)		13 (28.3)	8 (14.8)	
	pT4	25 (25.0)	11 (50.0)	14 (17.9)		18 (39.1)	7 (13.0)	
pN	pN0	49 (53.8)	10 (47.6)	39 (56.7)	0.514	17 (38.6)	32 (68.1)	**0.005**
	pN+	42 (46.2)	11 (52.4)	31 (44.3)		27 (61.4)	15 (31.9)	
pTNM stage	I	21 (21.0)	0 (0.0)	21 (26.9)	**0.008**	4 (8.7)	17 (31.5)	**0.008**
	II	18 (18.0)	3 (13.6)	15 (19.2)		6 (13.0)	12 (22.2)	
	III	17 (17.0)	3 (13.6)	14 (17.9)		9 (19.6)	8 (14.8)	
	IV	44 (44.0)	16 (72.7)	28 (35.9)		27 (58.7)	17 (31.5)	
PNI	0	69 (69.0)	9 (40.9)	60 (76.9)	**0.001**	23 (50.0)	46 (85.2)	**0.0002**
	1	31 (31.0)	13 (59.1)	18 (23.1)		23 (50.0)	8 (14.8)	
LVI	0	71 (71.0)	15 (68.2)	56 (71.8)	0.742	28 (60.9)	43 (79.6)	**0.039**
	1	29 (29.0)	7 (31.8)	22 (28.2)		18 (39.1)	11 (20.4)	
POI	1–3	46 (46.0)	3 (13.6)	43 (55.1)	**0.001**	8 (17.4)	38 (70.4)	**<0.0001**
	4, 5	54 (54.0)	19 (86.4)	35 (44.9)		38 (82.6)	16 (29.6)	
Adjuvant therapy	No	52 (52.0)	6 (27.3)	46 (59.0)	**0.009**	17 (37.0)	35 (64.8)	**0.005**
	Yes	48 (48.0)	16 (72.7)	32 (41.0)		29 (63.0)	19 (35.2)	

* Significant results are indicated in bold.

**Table 3:** Analysis of agreement between tumor–stroma ratio and tumor-infiltrating lymphocytes evaluated in biopsies and matched resection specimens.

		Biopsies			
		High TSR	Low TSR	Cohen’s Kappa (95% CI)	% of Agreement (95% CI)
**Resections**	High TSR	73 (73.0%)	0 (0.0%)	0.713 (0.551–0.875)	90.0% (82.4–95.1%)
	Low TSR	10 (10.0%)	17 (17.0%)		
		Low TILs	High TILs		
	Low TILs	19 (19.0%)	27 (27.0%)	0.372 (0.210–0.533)	70.0% (61.0–79.0%)
	High TILs	3 (3.0%)	51 (51.0%)		

**Table 4:** Univariate and multivariate Cox regression analysis for disease-free survival (DFS), disease-specific survival (DSS), and overall survival (OS) for tumor–stroma ratio (TSR) and tumor-infiltrating lymphocytes (TILs) in biopsies and resections, and other prognostic factors.

		DFS			DSS			OS		
		HR	95% CI	*p*-Value *	HR	95% CI	*p*-Value	HR	95% CI	*p*-Value *
Univariate analysis										
Age		0.993	0.968–1.018	0.566	0.978	0.944–1.013	0.210	0.991	0.964–1.019	0.536
Sex	Male	1			1			1		
	Female	1.345	0.762–2.375	0.306	1.435	0.651–3.165	0.370	1.040	0.538–2.010	0.907
Grade	1	1		**0.005**	1		**0.027**	1		**0.002**
	2	2.757	1.060–7.168	**0.038**	6.644	0.873–50.59	0.068	5.065	1.186–21.63	**0.028**
	3	4.856	1.804–13.07	**0.002**	12.83	1.652–99.56	**0.015**	10.80	2.501–46.67	**0.001**
pT	1	1		**0.0003**	1		0.291	1		**0.001**
	2	1.452	0.572–3.683	0.432	-	-	0.912	2.002	0.670–5.978	0.214
	3	4.314	1.816–10.25	**0.001**	-	-	0.906	6.627	2.374–18.50	**0.0003**
	4	4.306	1.863–9.951	**0.001**	-	-	0.905	4.515	1.601–12.74	**0.004**
pN	pN0	1			1			1		
	pN+	3.283	1.810–5.955	**<0.0001**	4.557	1.909–10.88	**0.001**	2.694	1.408–5.152	**0.003**
pTNM stage	I	1		**0.0003**	1		0.055	1		**0.006**
	II	0.826	0.233–2.927	0.767	-	-	0.896	0.947	0.254–3.527	0.935
	III	2.645	0.940–7.440	0.065	-	-	0.884	2.384	0.756–7.520	0.138
	IV	4.432	1.845–10.65	**0.001**	-	-	0.875	3.862	1.472–10.13	**0.006**
PNI	Absent	1			1			1		
	Present	3.949	2.251–6.927	**<0.0001**	6.249	2.792–13.99	**<0.0001**	4.006	2.137–7.511	**<0.0001**
LVI	Absent	1			1			1		
	Present	3.131	1.790–5.477	**<0.0001**	5.439	2.463–12.01	**<0.0001**	3.273	1.770–6.051	**<0.0001**
POI	1–3	1			1			1		
	4, 5	4.957	2.575–9.541	**<0.0001**	10.44	3.113–35.02	**0.0001**	5.238	2.473–11.09	**<0.0001**
Adjuvant therapy	No	1			1			1		
	Yes	3.197	1.788–5.718	**<0.0001**	6.799	2.545–18.16	**0.0001**	3.294	1.714–6.333	**0.0004**
Local recurrence	No	1			1			1		
	Yes	8.440	4.639–15.358	**<0.0001**	6.629	3.839–24.15	**<0.0001**	3.561	1.911–6.637	**<0.0001**
Margin	≥5 mm	1		**0.001**	1		**0.027**	1		**0.018**
	1–4.9 mm	0.690	0.358–1.331	0.268	0.864	0.343–2.179	0.757	0.754	0.368–1.547	0.442
	<1 mm	2.478	1.253–4.989	**0.009**	2.931	1.123–7.651	**0.028**	2.327	1.074–5.045	**0.032**
TSR resection	High TSR	1			1			1		
	Low TSR	5.087	2.859–9.053	**<0.0001**	6.555	2.940–14.62	**<0.0001**	5.504	2.875–10.54	**<0.0001**
TSR biopsy	High TSR	1			1			1		
	Low TSR	3.687	1.995–6.814	**<0.0001**	4.909	2.195–10.98	**0.0001**	3.542	1.778–7.056	**0.0003**
TILs resection	High	1			1			1		
	Low	3.824	2.121–6.897	**<0.0001**	3.879	1.676–8.975	**0.002**	4.010	2.061–7.802	**<0.0001**
TILs biopsy	High	1			1			1		
	Low	2.420	1.332–4.398	**0.004**	2.759	1.217–6.251	**0.015**	2.261	1.144–4.469	**0.019**
Multivariate analysis										
TSR resection ^&^	High TSR	1			1			1		
	Low TSR	4.403	2.241–8.648	**<0.0001**	5.010	2.032–12.35	**0.0005**	4.104	2.000–8.420	**0.0001**
TSR biopsy ^&^	High TSR	1			1			1		
	Low TSR	2.766	1.393–5.492	**0.004**	2.921	1.220–6.994	**0.016**	2.171	1.040–4.530	**0.039**
TILs resection ^&^	High TILs	1			1			1		
	Low TILs	2.118	1.043–4.302	**0.038**				2.210	1.022–4.776	**0.044**

^&^ adjusted for PNI, lymph node metastasis, grade, and pT. * Significant results are indicated in bold.

## Data Availability

The data presented in this study are available on request from the corresponding author. The data are not publicly available due to privacy and ethical restrictions.
